# Global research trends and hotspots on smoking and lung cancer from 1994–2023: A bibliometric analysis

**DOI:** 10.18332/tid/191857

**Published:** 2024-08-28

**Authors:** Yangfan Xu, Jieqiong Qi, Jiayao Liu, Yitao Jia

**Affiliations:** 1 Department of Oncology, Hebei General Hospital, Shijiazhuang, China; 2 Department of Medicine, Hebei North University, Zhangjiakou, China

**Keywords:** smoke, lung cancer, bibliometrics, visual analysis

## Abstract

**INTRODUCTION:**

Lung cancer is a significant cause of mortality, especially among smokers. Lung cancer and smoking are strongly associated, according to numerous studies.

**METHODS:**

Publications related to smoking and lung cancer were retrieved from the Science Citation Index Expanded (SCIE) database of the Web of Science Core Collection for the period 1994–2023. Descriptive and visual analyses were performed on the topics, journals, countries, institutions, authors, and citations of the publications.

**RESULTS:**

A total of 728 articles were retrieved from the Web of Science (WOS) SCIE database for the period January 1994 to December 2023, and the number of publications in the relevant literature demonstrated a progressive increase with time. A total of 647 articles were classified as experimental, while 81 were classified as reviews. The studies were published in 200 journals. The three journals that published the most articles were the American Journal of Epidemiology with 82 articles, Lung Cancer with 34 articles, and Cancer Causes and Control with 26 articles. The three most prolific countries were the United States (286 articles, 38.3%; 15879 citations), China (116 articles, 15.9%; 2911 citations), and France (75 articles, 10.3%; 3694 citations). The four most popular keywords in this field are ‘early cancer detection’, ‘experimental’, ‘CT’, and ‘survival rate’.

**CONCLUSIONS:**

The findings of our study revealed key areas for focus in smoking and lung cancer research, having a view of supplying important data and motivation for further investigations.

## INTRODUCTION

According to reports, in 2015 there were an estimated 17.5 million new cancer cases and 8.8 million cancer deaths worldwide^1^. In the year 2018, there was a global estimate of 18 million newly diagnosed cancer cases and 9 million deaths caused by cancer^2^. In 2020, there were an estimated 19.3 million cases of cancer worldwide, leading to nearly 10 million deaths^3^. Lung cancer is the primary cause of cancer-related fatalities worldwide, with a conservative approximation of 1.6 million deaths attributed to lung cancer annually. Recently, there has been a notable rise in the prevalence and fatality rates of lung cancer due to the growing number of smokers and the escalation of air pollution^4^. Cigarette smoke exhibits pro-inflammatory and immunosuppressive properties^5^, thereby elevating the susceptibility of smokers to cardiovascular disease, respiratory disease, rheumatoid arthritis, and other chronic ailments. Additionally, it poses a threat to non-smokers by exposing them to secondhand smoke, consequently heightening their vulnerability to chronic diseases and premature mortality^6,7^. At the cellular level, exposure to cigarette smoke exposure causes DNA damage^8^ and affects the frequency of DNA mutations^9-11^. Because of the delayed initial symptoms of lung cancer, the majority of patients are diagnosed at advanced stages. Chemotherapy and radiotherapy are still the two main therapies used for patients with advanced-stage lung cancer, which has limited effectiveness and accuracy^12^. Even with comprehensive treatment, patients have a relatively short survival time, with a five-year survival rate of less than 20%, indicating a poor prognosis^3,13^. Cigarettes contain a large number of carcinogenic substances, including nitrosamines, polycyclic aromatic hydrocarbons (PAHs) and other volatile organic compounds (VOCs), of which PAHs and nitrosamines have been shown to have a potent risk of causing lung cancer^14,15^. Gaining deeper insights into the patterns and developments in the realm of smoking and lung cancer investigation is of utmost importance for the prevention and management of lung cancer.

Numerous studies have conducted research on the association between smoking and lung cancer. However, the wide range of these studies often hinders researchers from identifying a suitable starting point. The existing relevant studies primarily concentrate on early detection of lung cancer, imaging tests for lung cancer patients, laboratory analyses, and investigations on survival rates. In addition, it has been noted that early smoking cessation is an effective measure to prevent lung cancer^16^. Hence, it is imperative to succinctly summarize the study material in this topic from a scientific standpoint.

Bibliometrics is a field that uses mathematical and statistical methods to analyze and quantify the influence and productivity of a certain field of research in various countries, organizations, journals, and by authors^17^. Bibliometric studies have been utilized to offer a lucid depiction of publication attributes, focal points, and research patterns in a certain discipline, with the aim of informing policy formulation^18^. This study employs bibliometric approaches to assess the literature on smoking and lung cancer that has been published by Web of Science over the course of the last thirty years. Our objective was to uncover the research findings, identify the areas of active research, and explore the cutting-edge topics in this field. Additionally, we analyzed the growth trajectory of the field based on the volume of published literature. The objective of this study is to provide significant insights to researchers in this subject and propose relevant directions for further study.

## METHODS

The data for this bibliometric study were sourced only from the Science Citation Index Expanded (SCIE) database, which is part of the Web of Science Core Collection (WoSCC). The WoSCC is acknowledged as a bibliographic resource of excellent quality, extensively embraced by researchers, and now the optimal database for conducting bibliometric research^19^. To ensure the accuracy and effectiveness in what was collected, SCIE was used for indexing, and the search technique was as follows: #1,[TS=(‘Lung Neoplasms’) OR TS=(‘Pulmonary Neoplasms’) OR TS=(‘Lung Neoplasm’) OR TS=(‘Pulmonary Cancer’) OR TS=(‘Pulmonary Cancers’) OR TS=(‘Cancer of the Lung’) OR TS=(‘Cancer of the Lung’)]; #2,[TS=(‘Smoking Behaviors’) OR TS=(‘Smoking Behavior’) OR TS=(‘Smoking Habit’) OR TS=(‘Smoking Habits’) OR TS=(‘Smoke’) OR TS=(‘Smoking’)]; #3, ‘#1’ AND ‘#2’. TS is an acronym for ‘Topic Subject’ search in WOS search. It is a method of searching data using topic phrases, based on Boolean logic. This approach allows for quick and easy retrieval of a large amount of material related to a certain issue. The search period was defined as 1994–2023. The data acquired from the WOS Core Collection comprises the title, keywords, publication date, authors, institution, country or area, journal, and total citation count. To minimize the impact of bias caused by frequent updates to the database, each of the literature searching along with information downloads were performed on the same day, specifically on 28 March 2024. The WOS database serves as the unique depository of all data in this study and does not necessitate ethical informed permission since it does not involve any human participants. The results of research on smoking and lung cancer were visualized and analyzed in this study using assessment and objective bibliometrics. In objective bibliometrics, the number of publications, citations, and citation analysis were all counted^20^. Number of publications (NP) and number of citations (excluding self-citations) (NC) were used to express productivity and impact, respectively. The contributions of nations, writers, journals, institutions, and their quantitative indicators, such as the h-index, as well as their quantitative values are assessed by evaluative bibliometrics^21,22^. Through this approach, articles that impact a field’s history can be found, along with current research hotspots and emerging trends^23,24^. Furthermore, the worth of an article is also demonstrated by its impact factor (IF) as indicated in the latest edition of the Journal Citation Reports (JCR)^25,26^.

### Inclusion and exclusion criteria

The inclusion criteria for the study were as follows: 1) The literature had to be in the form of essays or reviews; 2) The language used had to be English; and 3) The topic had to be closely associated with smoking and lung cancer.

The exclusion criteria consist of the following: 1) Any literature that is not in the form of abstracts of meetings, editorials, or letters; 2) Text documents that are not written in English; 3) Research that has been published before; and 4) Research that is not relevant to the issue of the study.

**Figure 1:**
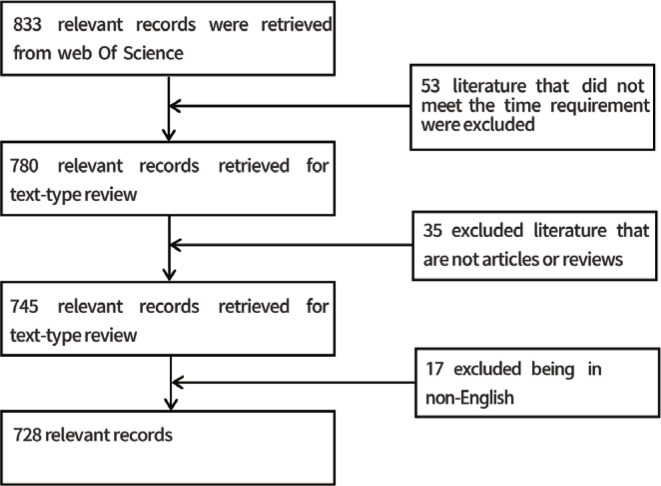
Flow diagram of research selection and screening

The articles that were obtained were subjected to screening, and the detailed procedure is depicted in Figure 1.

### Research methods

Analysis of data and visualization of literature was performed using CiteSpace 6.2.7.0 and VOSviewer 1.6.10, which are widely used visualization tools in bibliometrics to construct and analyze publication networks and trends in a given research area^27,28^. CiteSpace is a highly effective information visualization tool developed by Chen^29^. It is a free JAVA application for literature visualization and analysis of trends and patterns in the literature. VOSviewer is a software application created by Leiden University’s Center for Scientific and Technological Research. Its purpose is to simplify the creation and assessment of bibliometric networks^30^. The bibliometric analytic tools used in this study provided an unbiased and thorough perspective. Furthermore, we utilized an online bibliometric platform to examine the patterns of yearly publication and citation volume across various countries or regions. Additionally, employing VOSviewer co-occurrence analysis, we classified the terms extracted from the literature into distinct clusters and assigned different colors to them based on their chronological appearance. An examination of the co-occurrence of institutions and journals, as well as an analysis of keyword convexity, was conducted using CiteSpace. Furthermore, the software successfully identified the most significant spikes in citations for institutions, journals, and keywords.

## RESULTS

### Global trends in the number of publications

We initially retrieved 833 articles by performing the above search strategy on the literature, leaving 780 articles after excluding 53 articles that did not meet the time requirement. Subsequently, we further excluded 35 articles that did not meet the type of literature requirement, and included only 745 articles of the dissertation and review type. We eventually excluded another 17 non-English articles and included 728 documents in the bibliometric analysis. Figure 1 depicts the selection procedure.

The study analyzed a total of 728 publications, which were authored by 196 individuals affiliated with 185 institutions throughout 58 countries. These articles were published in a total of 200 periodicals. Supplementary file Figure 1 illustrates a polynomial regression curve representing the trend of the overall annual increase of relevant articles. The curve shows an increasing tendency in the number of articles annually that is positively connected with the period published (R^2^=0.5768). As demonstrated in Supplementary file Figure 2, the number of articles in the domain of smoking and lung cancer has consistently exhibited an annual upward trajectory from 1994 to 2023.

### Authors

The top decile of writers, based on publication count, collectively contributed 157 articles, or 21.57% of the overall publication count (Supplementary file Table 1). P. Bofita, a researcher from the International Agency for Cancer (IARC) in France, has the most extensive body of published work in the area of smoking and lung cancer. M.T. Randi from the National Institutes of Health (USA) and J. Siemiatycki from the University of Montreal (Canada) follow. H.-E. Wichmann from the Helmholtz Association in Germany, is an author who is heavily cited. Most of the top ten authors are from the Czech Republic, Canada and Germany.

### Journals

**Figure 2:**
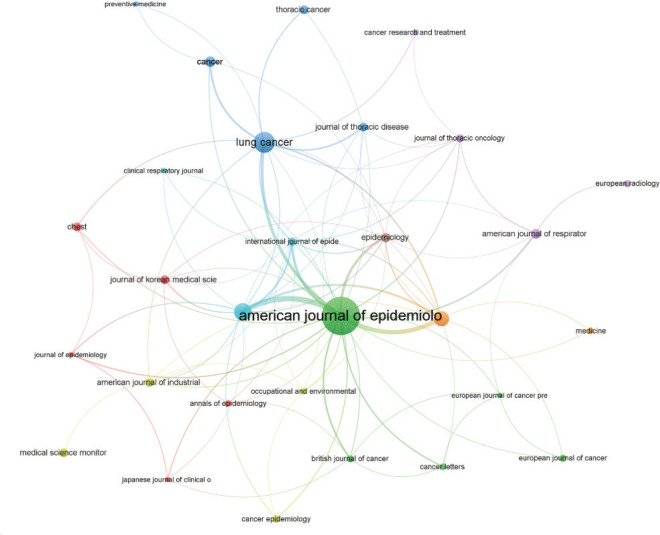
Citation analysis of published journals. Lines connect journals, whereas nodes symbolize them. Node size and connecting line thickness are exactly proportionate to journal citations and relevancy, respectively

The top ten journals, ranked by the number of articles published on smoking and lung cancer, collectively published 229 articles, representing 31.5% of the whole literature (Supplementary file Table 2). We acquired recent impact factors (IFs) of the pertinent journals by consulting Journal Citation Reports (JCR) 2022. We utilized the impact factor as a primary criterion to evaluate the scholarly influence of the publications. The top five journals included *American Journal of Epidemiology* (82 articles, 11.3%), *Lung Cancer* (34 articles, 4.7%), *Cancer Causes Control* (26 articles, 3.6%), *International Journal of Cancer* (21 articles, 2.9%), and *Cancer* (13 articles, 1.8%). The analysis of journals for citations was conducted using VOSviewer, as depicted in Figure 2. The size of the nodes in the figure represents the number of citations. The American Journal of Epidemiology, Cancer Causes Control, Lung Cancer, and International Journal of Cancer were identified as highly cited journals and were positioned at the core of the citation network. The American Journal of Epidemiology stands out among other journals due to its significantly higher number of published articles and the regularity with which its publications are cited.

### Countries

The United States had the largest number (286; 39.3%) of published articles, then China (116; 15.9%), France (75; 10.3%), Canada (66; 9.1%), the United Kingdom (56; 7.7%), Germany (47; 6.5%), South Korea (47; 6.5%), Spain (47; 6.5%), Italy (46; 6.3%), and Switzerland (46; 6.3%). Table 1 presents a hierarchical arrangement of the leading 10 countries, determined by the overall number of published articles. The nations with the largest number of citations were the United States (15978), France (3964), Canada (3039), China (2911), and the United Kingdom (2714). The USA had the largest number of published articles and citations. We performed a study to assess the extent of collaboration among countries in this specific subject. To represent the collaborative relationships between countries, we utilized VOSviewer. The size of the nodes in Supplementary file Figure 3 corresponds to the number of publications, whereas the connections between nodes show direct collaboration relationships among countries. Research indicates that England and Germany maintain stronger international relationships, positioning them as key hubs for global collaboration.

**Table 1:** Top ten countries and citation frequency of smoking and lung cancer literature published in WOS database

*Rank*	*Country*	*NP*	*NC*	*H-index*	*Average citation per item*
1	USA	286	15978	70	56.59
2	CHINA	116	2911	28	25.36
3	FRANCE	75	3964	34	53.21
4	CANADA	66	3039	34	46.47
5	ENGLAND	56	2724	28	49.02
6	GERMANY	47	2004	27	43.17
7	SOUTH KOREA	47	1416	18	30.47
8	SPAIN	47	1341	22	29.15
9	ITALY	46	1994	25	43.63
10	SWEDEN	46	2637	28	57.89

NP: number of publications. NC: number of citations.

Supplementary file Figure 5 shows the number of publications per year for the most influential countries, with the size and color of the circles corresponding to the number of publications. The United States has been at the center of global research in the area of smoking and lung cancer, while China has been more prolific in recent decades. Supplementary file Figure 6 depicts the global distribution of publications by country/region, and the results indicate that smoking and lung cancer-related research is unevenly distributed globally.

### Organizations

Globally, the National Institutes of Health (NIH) (52 articles; 7.14%) is the organization with the most publications in this field in the last 30 years, with 2913 citations to its articles. Table 2 illustrates that among the top 10 universities ranked by the number of published articles, four were from the United States, two from France, and the other institutions were from Germany, Switzerland, Spain, and the United Kingdom. We utilize VOSviewer to graphically depict and analyze the collaborative relationships between institutions, thereby uncovering the essence of these collaborations (Supplementary file Figure 4).

**Table 2:** Top ten institutions in published studies related to smoking and lung cancer

*Rank*	*Affiliation*	*NP*	*NC*	*Country*	*H-index*	*Average citations per item*
1	NATIONAL INSTITUTES OF HEALTH	52	2913	USA	31	56.42
2	WORLD HEALTH ORGANIZATION	45	2704	FRANCE	28	60.6
3	HARVARD UNIVERSITY	35	1990	USA	22	57
4	INSTITUT NATIONAL DE LA SANTE ET DE LA RECHERCHE MEDICALE INSERM	33	1683	FRANCE	22	51.3
5	HELMHOLTZ ASSOCIATION	32	1353	GERMANY	22	42.75
6	UNIVERSITY OF CALIFORNIA SYSTEM	30	3000	USA	23	100.07
7	KAROLINSKA INSTITUTET	29	2063	SWEDEN	24	71.66
8	UNIVERSITY OF TEXAS SYSTEM	26	1075	USA	16	41.46
9	CIBER CENTRO DE INVESTIGACION BIOMEDICA EN RED	24	487	SPAIN	12	21
10	UNIVERSITY OF LONDON	23	1002	ENGLAND	17	43.78

### Keywords

**Figure 3:**
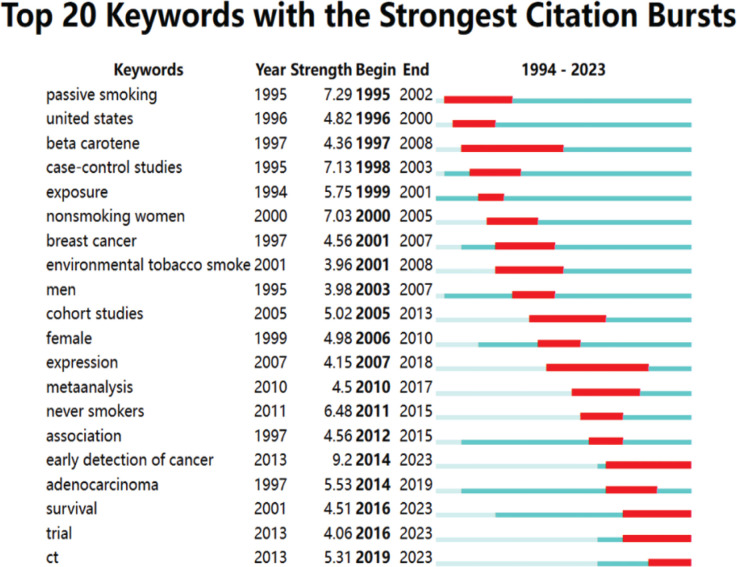
The 20 most often occurring keywords in the context of smoking and lung cancer. The blue line depicts the history, while the red section reflects the specific time period in which the outbreak of the keyword took place, including the initial year, final year, and duration of the outbreak

A total of 86 keywords, which occurred with a frequency of ≥5, were selected from the abstracts and titles of the 728 articles that were assessed. VOSviewer was then utilized to evaluate the simultaneous appearance of these keywords. In Supplementary file Figure 7, the 86 keywords were categorized into 10 categories: category 1 (studies related to early detection of lung cancer, red), category 2 (studies related to the effects of environmental pollution on cancer, green), category 3 (studies related to the effects of smoking on carcinogenesis, blue), category 4 (studies related to the diagnosis of lung cancer, yellow), category 5 (studies related to the effects of dietary habits on lung cancer, purple), category 6 (studies related to the effect of occupational exposure on lung cancer, cyan), category 7 (studies related to lung diseases, orange), category 8 (studies related to baseline information of the diseased population, brown), category 9 (studies related to non-small cell lung cancer, pink), and category 10 (the effect of genetic factors on factors related to the development of cancer, light pink), and the size of the nodes corresponds to the rate with which the keyword occurs. The nodes were assigned distinct colors based on the chronological occurrence of keywords (Supplementary file Figure 8). The predominant time frame during which high-frequency keywords emerged was primarily from 2006 to 2011. The color dark blue represents keywords that were mentioned earlier, whereas the color yellow represents keywords that were mentioned more recently. In order to gain a deeper understanding of the current areas of focus in the study of smoking and lung cancer, we utilized the burst analysis feature of CiteSpace to examine the articles published on this topic between 1994 and 2023. As a result, we identified 20 keywords that are particularly significant in this research domain. Burst words are highly recurrent keywords during a particular timeframe. These findings not only illustrate the changing trends in study focus over time, but also indicate the current areas of interest and offer recommendations for future research initiatives. As shown in Figure 3, ‘passive smoking’ (strength 7.29), ‘case control study’ (strength 7.13), ‘exposure’ (strength 5.75), ‘non-smoking women’ (strength 7.03), and ‘never smokers’ (strength 6.48) were the top topics in the field between 1995–2013. Over the next 10 years, ‘early detection of cancer’ (strength 9.2), ‘survival rate’ (strength 4.51), ‘trial’ (strength 4.06) and ‘CT’ (strength 5.31) are gradually coming to the forefront of research on smoking and lung cancer.

### Co-citation analysis

We performed a rating of the 728 articles that were discovered, utilizing the frequency of citations as the determining factor. Subsequently, we generated a compilation of the foremost 10 that garnered the most citations (Supplementary file Table 3). The study with the most references is titled ‘The Changing Cigarette, 1950–1995’ by D. Hoffmann. It was published in 1997 in the Journal of Toxicology And Environmental Health.

**Figure 4:**
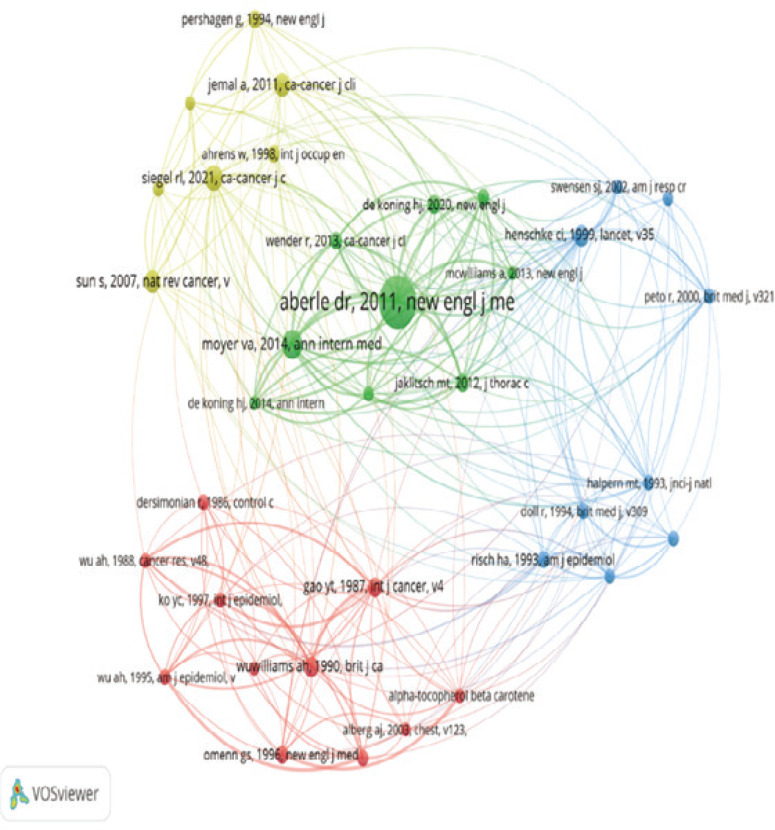
A co-citation map related to smoking and lung cancer. The nodes represent articles, while the lines indicate the links between them. The frequency of co-citations of the article is directly proportional to the size of the nodes, while the thickness of the connecting line is directly proportionate to the degree of relevance

The literature in question has total citations of 653, with an average annual citation frequency of 23.32. Furthermore, the study titled ‘Pulmonary Oxidative Stress, Inflammation and Cancer: Respirable Particulate Matter, Fibrous Dusts and Ozone as Major Causes of Lung Carcinogenesis through Reactive Oxygen Species Mechanisms’ by A. Valavanidis was published in 2010 in the International Journal of Environmental Research and Public Health and was listed in the top ten based on its average annual citation frequency of 42.8. However, the total number of citations for this article was 505. The co-citation analysis was conducted using VOSviewer, revealing that among the 36 documents, each document received ≥15 citations. The article titled ‘Reduced Lung-Cancer Mortality with Low-Dose Computed Tomographic Screening’ was particularly prominent in the co-citation pattern, as seen in Figure 4.

## DISCUSSION

According to the SCIE database in the Web of Science core collection, this study conducted a comprehensive literature review on the relationship between smoking and lung cancer, focusing on publications up to December 2023. It employed bibliometric visualization analysis to acquire a comprehensive comprehension of the present research landscape in this domain. The objective was to identify research trends and areas of focus, and to offer guidance for future research endeavors.

Through an analysis of the findings and citations in scholarly literature, one can effectively assess the academic reputation and influence of countries, institutions, and scholars in a specific field. This analysis helps to uncover the specific roles they play in the field. The survey reveals a consistent upward trajectory in the number of research articles published on the topic of smoking and lung cancer, with time. Over the past several years, there has been a steady increase in the number of articles published on this specific issue. It is projected that the number of articles will reach its peak in 2020, highlighting the fact that this research field has gained significant global attention

Concerning the number of published works and the measure of their influence, American Journal of Epidemiology, Lung Cancer, and Cancer Causes Control are the major journals that publish relevant articles.

The United States, China, and France are globally recognized for their significant contributions to literature, with the United States, France, and Canada being particularly renowned for their highly cited works and substantial academic influence. China has made a significant contribution to the field by publishing a comparatively high number of articles, constituting a substantial proportion of the total publications. The United States maintains the strongest academic partnerships with other nations, and as global cultural integration progresses, there will be an increase in joint studies among scholars from many countries in the future.

When it comes to the number of articles and citations produced by institutions, four of the top 10 institutions with the highest output are in the United States, while the other six are all in Europe. This pattern aligns with their dominant role in the field of smoking and lung cancer research. Among them, the National Institutes of Health (NIH) in the United States published a significant proportion of the total articles, ranking first among all institutions. The NIH’s substantial contributions to the topic of smoking and lung cancer have been generally acknowledged by international researchers. The other four institutions with the highest number of citations are the University of California, the World Health Organization, the Karolinska Institutet, and Harvard University. Among them, the University of California and Harvard University are located in the United States, while the World Health Organization and the Karolinska Institutet are located in Europe, which is proof of the status of the United States and Europe in the field of smoking and lung cancer research.

The latest burst words are ‘early detection of cancer’, ‘trial’, ‘survival rate’ and ‘CT’. It can be seen that with the deepening of previous research in the field of smoking and lung cancer, more and more scholars have begun to pay attention to the importance of early diagnosis of lung cancer to improve the survival rate. Among them, imaging and laboratory tests are indispensable ways to confirm the diagnosis of lung cancer. Promptly quitting smoking through various means is of utmost significance in preventing or advancing lung cancer, and can additionally enhance the living level for patients.

### Strengths and limitations

This investigation performed an extensive and methodical search of the WOS database to impartially examine the advancements in studies on smoking and lung cancer throughout the last three decades. The text recognized the valuable contributions made by writers, nations, academic institutions, periodicals, and other entities. Furthermore, it employed bibliometrics to examine and forecast upcoming areas of study interest. The study also provided several reference recommendations for future study endeavors in this specific topic.

However, certain limitations are still unavoidable. Initially, the bibliometrics program has inherent limitations. Furthermore, this study solely examined the WOS SCIE database and did not encompass pertinent material from alternative databases. Furthermore, this study specifically focused on analyzing articles written in English, which led to the deliberate omission of potentially relevant literature written in other languages. Furthermore, only scholarly articles and reviews were considered for inclusion, while conference publications and books, which could potentially have academic significance, were excluded. Ultimately, bibliometrics is just a quantitative analytical method without the capacity to fully and methodically evaluate the quality of published studies.

## CONCLUSIONS

Bibliometric analyses show that research on smoking and lung cancer is progressing rapidly and the literature in this area is increasing year by year. The American Journal of Epidemiology is the journal with the largest number of publications in this area. The United States has provided the most major contribution for the advancement in this field, as seen by the inclusion of four American universities in the top 10 rankings. The latest burst words are ‘CT’, ‘early detection of cancer’, ‘survival rate’ and ‘trial’. In the future, it is necessary to address several unresolved matters to thoroughly investigate the correlation among smoking and lung cancer, enhance the general health of those diagnosed with lung cancer, and improve their chances of survival.

## Supplementary Material



## Data Availability

All data for this study can be found in the WOS (https://www.Webofscience.com). The original contributions presented in the study are included in the article. Further inquiries can be directed to the corresponding author.
