# Supplementary material for: Global research trends and hotspots on smoking and lung cancer from 1994–2023: A bibliometric analysis
Source: Tob Induc Dis. 2024 Aug 28;22:10.18332/tid/191857. doi: 10.18332/tid/191857 (PMC11350636; doi:10.18332/tid/191857)
Supplement: Supplementary file 1 [file TID-22-148-s1.pdf]

SUPPLEMENTARY MATERIALS

Supplementary Tables

Supplementary Table 1. Top 10 authors with the most publications.

Rank	Author	NP	NC	Country/Region	Affiliation	H-index	Average citation per item
1	Boffetta, Paolo	29	1550	FRANCE	INTERNATIONAL AGENCY FOR RESEARCH ON CANCER IARC	23	54.14
2	Landi, Maria Teresa	17	591	USA	NATIONAL INSTITUTES OF HEALTH NIH	11	35.18
3	Siemiatycki, Jack	16	597	CANADA	Universite de Montreal	11	37.75
4	Ruano-Ravina, Alberto	15	408	SPAIN	Universidade de Santiago de Compostela	11	28.47
5	Zaridze, David	14	638	Czech Republic	Charles University Prague	12	46.43
6	Lissowska, Jolanta	14	638	Czech Republic	Charles University Prague	12	46.43
7	Janout, Vladimir	14	638	Czech Republic	Charles University Prague	12	46.43
8	Parent, Marie-Elise	13	635	CANADA	University of Quebec	12	49.15
9	Wichmann, Heinz Erich	13	806	GERMANY	Helmholtz Association	11	62.92
10	Ahrens, Wolfgang	12	710	GERMANY	Leibniz institute for Prevention Research & Epidemiology	10	59.92

Supplementary Table 2. Top ten productive journals related to smoke and lung cancer.

Rank	Journal	NP	NC	IF （2022）	H-index	Average citation per item
1	AMERICAN JOURNAL OF EPIDEMIOLOGY	82	6945	5	51	85.51
2	LUNG CANCER	34	1428	5.3	20	42.09
3	CANCER CAUSES CONTROL	26	1306	2.3	19	50.35
4	INTERNATIONAL JOURNAL OF CANCER	21	841	6.4	16	40.14
5	CANCER	13	531	6.2	10	41

6	AMERICAN JOURNAL OF RESPIRATORY AND CRITICAL CARE MEDICINE	12	1148	24.7	11	95.83
7	EPIDEMIOLOGY	11	680	5.4	11	62
8	AMERICAN JOURNAL OF INDUSTRIAL MEDICINE	10	439	3.5	8	44.1
9	CHEST	10	1105	10.1	9	110.6
10	JOURNAL OF KOREAN MEDICAL SCIENCE	10	207	4.5	8	21

Supplementary Table 3. Top ten cited literature related to smoke and lung cancer.

Rank	Title	type	Author	Journal	Publication Year	NC	Average per Year
1	The changing cigarette, 1950-1995	Review	Hoffmann, D	JOURNAL OF TOXICOLOGY AND ENVIRONMENTAL HEALTH	1997	653	23.32
2	Marked increase in bladder and lung cancer mortality in a region of Northern Chile due to arsenic in drinking water	Article	Smith, AH	AMERICAN JOURNAL OF EPIDEMIOLOGY	1998	587	21.74
3	American Cancer Society Lung Cancer Screening Guidelines	Article	Wender, R	CA-A CANCER JOURNAL FOR CLINICIANS	2013	540	45
4	Estimates of cancer incidence and mortality in Europe in 1995	Article	Bray, F	EUROPEAN JOURNAL OF CANCER	2002	536	23.3
5	Lung cancer screening with CT: Mayo Clinic experience	Article	Swensen, SJ	RADIOLOGY	2003	517	23.5
6	Pulmonary Oxidative Stress, Inflammation and Cancer: Respirable	Review	Valavanidis, A	INTERNATIONAL JOURNAL OF ENVIRONMENTAL RESEARCH AND PUBLIC HEALTH	2013	505	42.08

	Particulate Matter, Fibrous Dusts and Ozone as Major Causes of Lung Carcinogenesis through Reactive Oxygen Species Mechanisms						
7	The effects of waterpipe tobacco smoking on health outcomes: a systematic review	Review	Akl, EA	INTERNATIONAL JOURNAL OF EPIDEMIOLOGY	2010	430	28.67
8	Radiation-induced pulmonary toxicity: A dose-volume histogram analysis in 201 patients with lung cancer	Article	Hernando, ML	INTERNATIONAL JOURNAL OF RADIATION ONCOLOGY BIOLOGY PHYSICS	2001	388	16.17
9	A clinical model to estimate the pretest probability of lung cancer in patients with solitary pulmonary nodules	Article	Gould, MK	CHEST	2007	305	16.94
10	Long-Term Follow-up Results of the DANTE Trial, a Randomized Study of Lung Cancer Screening with Spiral Computed Tomography	Article	Infante, M	AMERICAN JOURNAL OF RESPIRATORY AND CRITICAL CARE MEDICINE	2015	268	26.8

Supplementary Figures

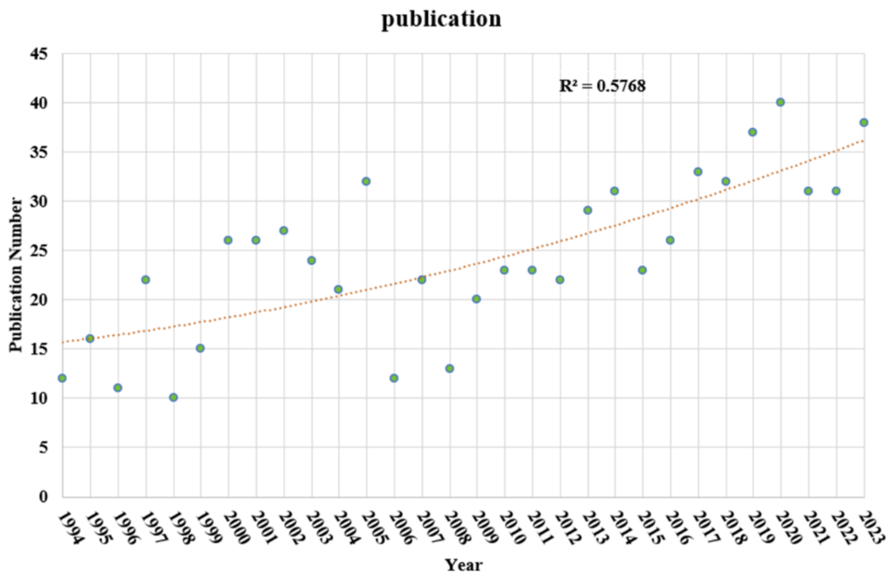

Supplementary Figure 1. Dot Plot of Annual Publications on Smoking and Lung Cancer, 1994-2023.

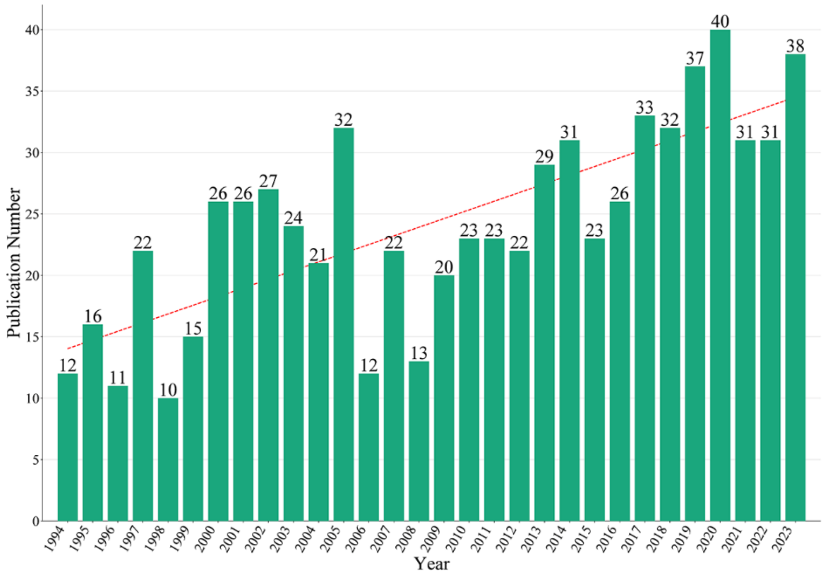

Supplementary Figure 2. Histogram of Annual Publications on Smoking and Lung Cancer, 1994-2023.

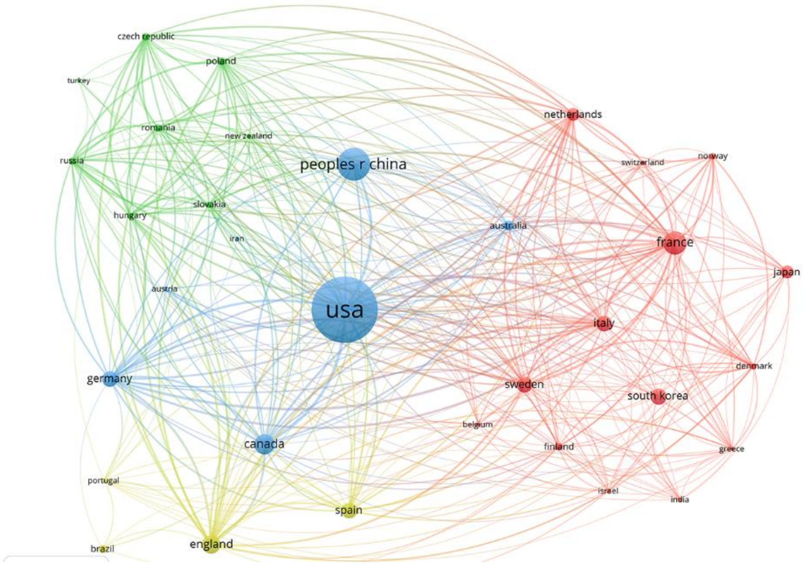

Supplementary Figure 3. Countries collaboration analysis. The nodes represent countries, and the lines indicate the connections between them. The size of each node correlates with the number of publications, while the thickness of the connecting lines correlates with the level of cooperation between the countries.

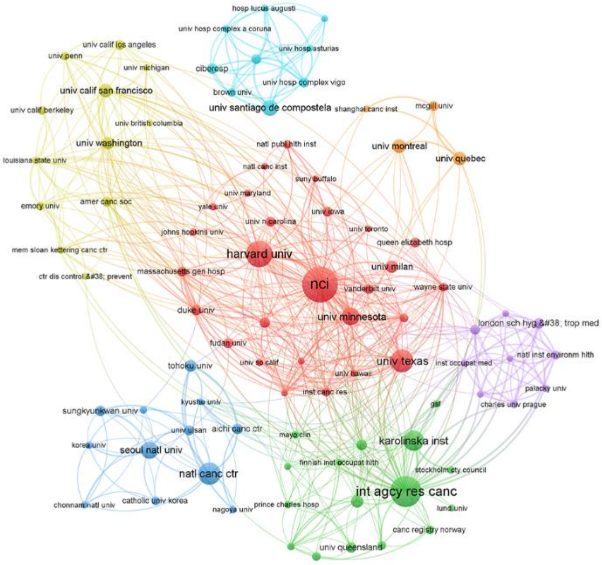

Supplementary Figure 4. Global institutions collaboration analysis. The nodes symbolize institutions, while the lines denote the connections between them. The publication number is proportional to the size of nodes, and the thickness of the connecting line is proportional to the degree of cooperation. The publication number is proportional to the size of nodes, and the thickness of the connecting line is proportional to the degree of cooperation.

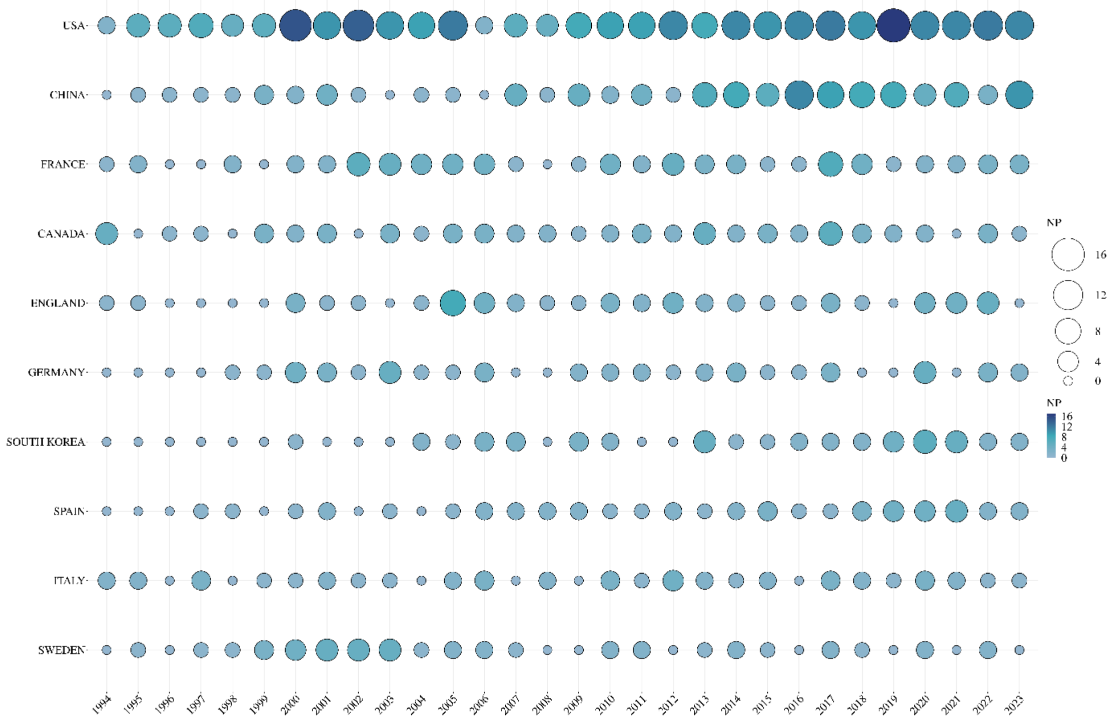

Supplementary Figure 5. Annual output trend of the top 10 productive countries; the size and color of the circles represent the number of publications (NP).

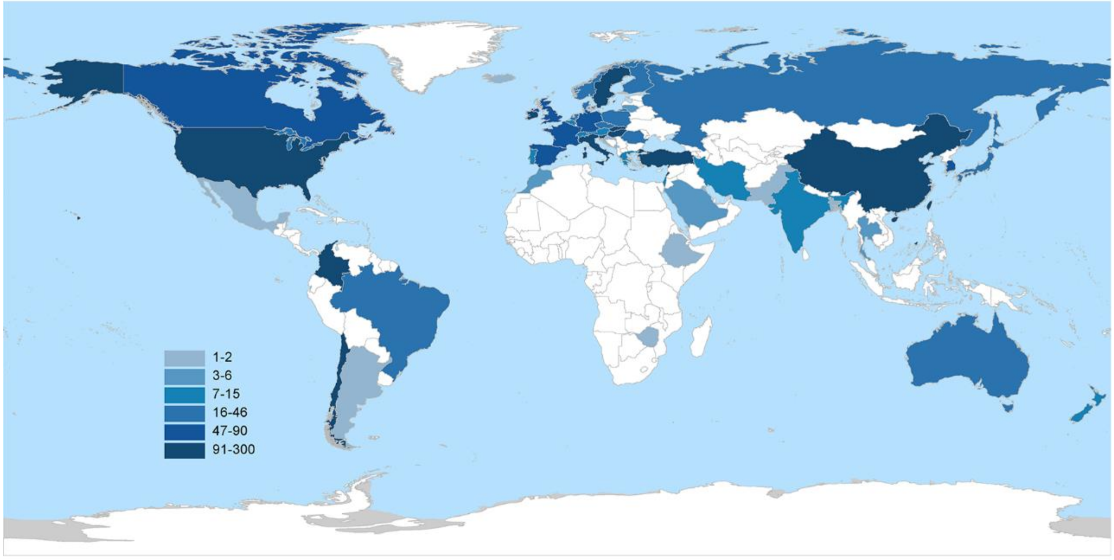

Supplementary Figure 6. Publication counts distribution per country/region.

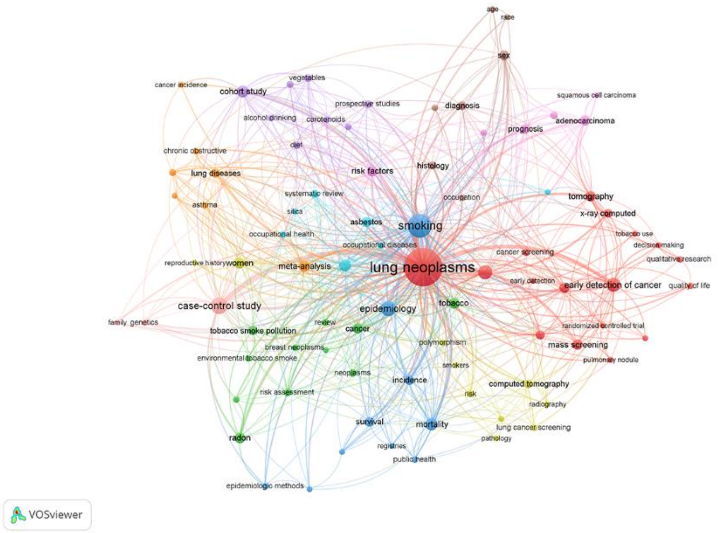

Supplementary Figure 7. The VOSviewer tool was used to create a network representation of keywords found in papers related to smoking and lung cancer. A compilation of the terms relevant to the specified location. The words were categorized into ten groups based on the default color scheme. Studies on the timely identification of lung cancer (red), studies on the impact of environmental pollution on cancer (green), studies on the correlation between smoking and cancer development (blue), research pertaining to the diagnosis of lung cancer (yellow), studies investigating the impact of food patterns on lung cancer (purple) have been conducted. Research investigating the impact of workplace exposure on lung cancer (cyan), studies examining lung illness (saffron), foundational research on information-related topics within the impacted population (brown), studies pertaining to non-small cell lung cancer (NSCLC), genetic variables have a significant impact on the onset and progression of cancer (light pink). The circle of significant magnitude symbolizes the keywords that have occurred with a notable frequency.

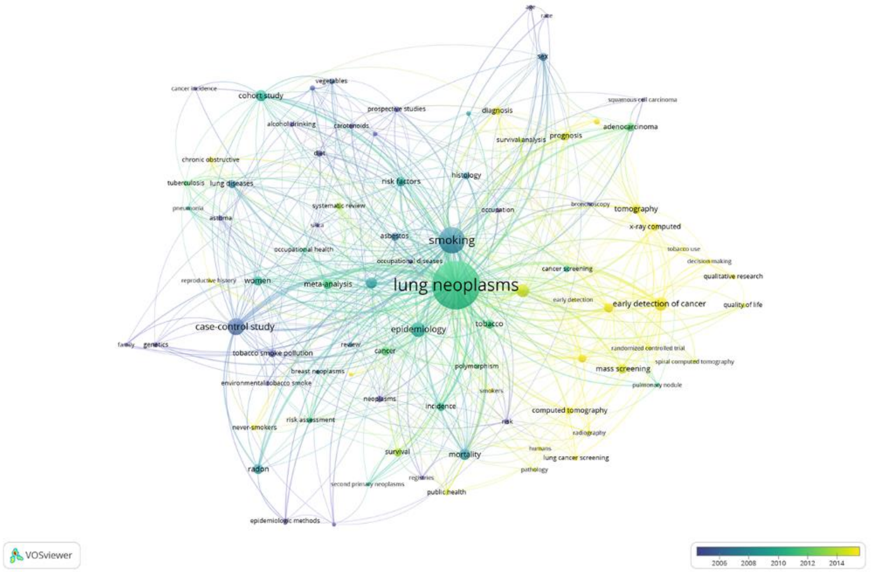

Supplementary Figure 8. The distribution of keywords was provided based on their average appearance time. Keyword bursts primarily concentrate on the time period between 2006 and 2011. The color blue signifies an initial occurrence, while the color yellow signifies a more recent occurrence. As the distance between two keywords decreases, their frequency of co-occurrence increases.

©2024 Xu Y. et al.
